# Molecular Mechanisms of Aging and Immune System Regulation in *Drosophila*

**DOI:** 10.3390/ijms13089826

**Published:** 2012-08-07

**Authors:** Ioannis Eleftherianos, Julio Cesar Castillo

**Affiliations:** Insect Infection and Immunity Lab, Department of Biological Sciences, Microbiology and Immunology Program, Institute for Biomedical Sciences, The George Washington University, Lisner Hall 336, 2023 G Street NW, Washington, DC 20052, USA; E-Mail: juliocastillo25@gmail.com

**Keywords:** aging, innate immunity, infection, insects, gene transcription, antimicrobial peptides, age-related diseases

## Abstract

Aging is a complex process that involves the accumulation of deleterious changes resulting in overall decline in several vital functions, leading to the progressive deterioration in physiological condition of the organism and eventually causing disease and death. The immune system is the most important host-defense mechanism in humans and is also highly conserved in insects. Extensive research in vertebrates has concluded that aging of the immune function results in increased susceptibility to infectious disease and chronic inflammation. Over the years, interest has grown in studying the molecular interaction between aging and the immune response to pathogenic infections. The fruit fly *Drosophila melanogaster* is an excellent model system for dissecting the genetic and genomic basis of important biological processes, such as aging and the innate immune system, and deciphering parallel mechanisms in vertebrate animals. Here, we review the recent advances in the identification of key players modulating the relationship between molecular aging networks and immune signal transduction pathways in the fly. Understanding the details of the molecular events involved in aging and immune system regulation will potentially lead to the development of strategies for decreasing the impact of age-related diseases, thus improving human health and life span.

## 1. Introduction

The process of aging involves changes in physiology, metabolism and reproduction in all eukaryotic organisms. This is due to the appearance of deleterious mutations, malfunction in certain components of proteolytic systems and continuous accumulation of intracellular damaged proteins that lead to different pathologies and eventually to reduced lifespan [1]. Previous studies using model organisms have uncovered genes with major effects on longevity. Such genes were mainly found to be involved in pathways regulating metabolic functions or in pathways controlling the response to environmental factors [2]. It has been proposed that mutations in genes that code for components of such regulatory pathways appear to have been evolutionarily derived to fine-tune the expression of genes predicted by the classical evolutionary theory of aging [3]. Eukaryotic organisms have evolved sensitive systems for detecting the invasion of microorganisms and efficient mechanisms for suppressing the growth of pathogens and eliminating them from the body [4]. The vertebrate immune system consists of innate immunity, which is the first line of host defense against foreign microbes, and adaptive, or acquired, immunity, which acts in the late phase of infection and is characterized by the generation and maintenance of immunological memory, and specificity that is provided by pathogen-specific receptors [5]. Invertebrates, however, lack antibody-mediated mechanisms for the destruction of infectious agents. Instead, they recognize microorganisms using germline-encoded Pattern Recognition Receptors (PRR), which trigger well-conserved signaling pathways that in turn lead to the activation of a sophisticated panel of immune responses. Innate immune mechanisms act locally or systemically and participate in multiple functions that efficiently fight off pathogenic infections [6]. It is now widely accepted that aging is characterized by a functional decline in immune defenses [7].

Research efforts in the areas of aging and immunity have been expanding exponentially in recent years because both fields have been favored from progress in molecular biology techniques as well as in many other research areas, ranging from cell biology and genetics to genomics, transcriptomics and proteomics. Furthermore, recent studies on the molecular basis of aging in laboratory model organisms are now expanding to humans, thus making possible a better understanding of the cause of age-related diseases [8]. Insects serve as outstanding research tools in several areas of biology and biomedicine for studying molecular/genetic mechanisms of key biological processes, including aging and immunity. Interest in insects as alternative experimental models has grown in previous years as researchers have taken into account society’s ethical concerns regarding the use of vertebrate animals in teaching, testing and laboratory research [9,10]. In particular, *Drosophila melanogaster*, with a vast number of molecular, genetics and genomics tools available, is widely recognized as an outstanding model organism for investigating complex traits in human biology. Research in *Drosophila* has led to the identification of important aging and immune signaling pathways, and the development of models for life span and several human diseases [11].

Molecular and genetics studies in *Drosophila* have recently started investigating the interplay between immunity and aging mechanisms. Here, we review the recent advances in our understanding of the molecular basis underlying aging and immunity and their interaction in *Drosophila*. Understanding the effect of aging on the immune response and *vice versa* as well as the interconnection between signaling pathways governing these major biological processes is critical for elucidating age-related functional decline in humans, which could lead to new therapeutic approaches that could potentially improve protection against disease in older individuals.

## 2. Molecular Mechanisms of Aging in *Drosophila*

A great deal has been learned about the molecular mechanisms that govern the aging process and its regulation in insects during the past decade. In several cases, aging research has led to the identification of certain genes or gene families with major effects on longevity [12]. These genes have been shown to modulate important factors of metabolic functions, in particular concerning energy-related functions of the organism, such as the insulin signaling pathway. Other pathways implicated in mitochondrial activities have also been reported to play a role in *Drosophila* aging and lifespan extension pathways [13].

TOR (Target of Rapamycin) is a protein kinase that regulates nutrient sensing, protein synthesis and metabolism to maintain homeostasis in eukaryotic organisms. Mutations in TOR genes slow down growth or development in various species. These phenotypes are equivalent to those found under poor nutritional conditions [14]. The TOR signaling pathway has been shown to play an important role in several human diseases, such as cancer, diabetes and heart dysfunctions, which affect various physiological processes that determine longevity [15]. *Drosophila* TOR (dTOR) is also involved in several biological processes, including maintenance of normal growth and development. Deficiency of dTOR function in *Drosophila* results in smaller size and numbers of cells in diverse fly tissues [16,17]. The role of dTOR in lifespan of *Drosophila* has been demonstrated by the fact that inhibition of dTOR pathway signaling by ubiquitous overexpression of a dominant-negative allele of dTOR produces a mean lifespan increase of 24%–26%. A similar result was also obtained after activation of the genes encoding Tuberous sclerosis 1 (dTsc1) and 2 (dTsc2) that act as negative regulators upstream of TOR in *Drosophila* [18]. In addition, it was previously shown that tissue-specific gene up-regulation can have a profound effect on fly longevity. Overexpression of dFOXO (forkhead box, subgroup “O”) in the fat body and brain, or overexpression of dTsc2 in the fat body and muscles of *Drosophila* transgenic flies, is sufficient to cause lifespan extension [18–20]. Interestingly, Sestrin, a highly conserved protein that accumulates in cells exposed to stress, was recently suggested to be involved as a negative feedback regulator of TOR that prevents age-associated pathological effects, such as triglyceride accumulation, mitochondrial damage, muscle deterioration, and heart function impairment in flies. These pathologies are probably induced by prolonged activation of the TOR pathway in *Drosophila* [21].

**Figure 1:**
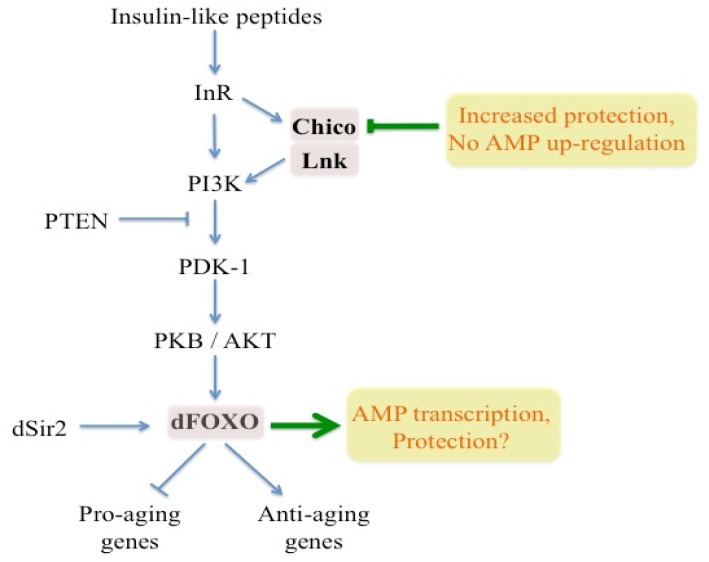
Interaction between the insulin/Igf-like (IIS) signaling pathway with immune function and pathogen resistance in *Drosophila*. Insulin Receptor (InR) is initially activated upon binding of insulin-like peptides. The activated receptor either phosphorylates directly the enzyme phosphoinositide 3-kinase (PI3K) or acts indirectly through the insulin receptor substrate proteins Chico and Lnk. PI3K is a lipid kinase consisting of the regulatory subunit dP60 and the catalytic subunit dP110. PI3K is then recruited to the membrane where it phosphorylates the 3′ position of phosphatidylinositol-(4,5)-bisphosphate (PIP_2_) and thereby generates phosphatidylinositol-(3,4,5)-trisphosphate (PIP_3_). Elevated levels of PIP_3_ recruit the PH-domain-containing protein kinase B (PKB/AKT) to the plasma membrane, facilitating its activation by phosphoinositide-dependent kinase 1 (PDK1). PKB signals downstream by inhibiting the *FOXO* (forkhead box, subgroup “O”) family of transcription factors (*dFOXO* in *Drosophila*). Inhibition of the IIS pathway allows *dFOXO* to negatively regulate the expression of pro-aging genes and positively regulate the expression of anti-aging genes. PTEN is a negative regulator of the IIS pathway. It is not currently clear whether overexpression of *Silent information regulator 2* (*Sir2* or *Sirtuin*) increases longevity in *Drosophila. Chico* mutant flies have improved survival after bacterial infection that is not due to AMP up-regulation. Transcription factor *dFOXO* regulates antimicrobial peptide (AMP) transcription but its activation does not protect flies against pathogenic infection.

The TOR pathway interacts extensively with the insulin/insulin-like growth factor (Igf) signaling pathway. In particular, limiting insulin/Igf-like signaling (IIS) inhibits TOR activity that results in lifespan extension (Figure 1) [22,23]. The effects of the IIS system on longevity of the adult fly have not been investigated in great detail thus far. Interestingly, it has been shown that *Drosophila* lifespan is prolonged when insulin-like peptides (mainly produced in the brain and fat body tissues), the insulin receptor and its substrates Chico and Lnk are down-regulated or when the negative IIS pathway regulator PTEN is up-regulated [20,24–27]. Decreased IIS signaling leading to increased lifespan has also been associated with increased resistance to oxidative stress as well as increased activity of cellular detoxification pathways [28,29]. Transcription of certain genes encoding *Drosophila* insulin-like peptides is reduced under dietary restriction conditions that increase fly lifespan [30,31]. However, deletion of insulin-like peptide genes results in higher lifespan at increased diet consumption levels [24]. Dietary restriction, which is the reduction of nutrient intake without causing malnutrition, has been previously linked to IIS and TOR signaling pathways. In particular, it has been found that reduced activity of the *Drosophila* TOR pathway promotes lifespan in a way that resembles the effects of dietary restriction on fly longevity [18], and feeding adult flies with the inhibitory drug rapamycin leads to increased longevity as the result of changes in autophagy and protein synthesis. Rapamycin also increased the lifespan of IIS pathway mutants and of flies subjected to dietary restriction [32]. In addition, dFOXO was found to be involved in the response to dietary restriction, because *dFOXO* gain-of-function mutants showed increased longevity at higher diet concentrations [33]. Reproductive capacity is strongly inhibited by reduced IIS/TOR signaling that also reduces the impact of aging-related pathologies, such as cardiac dysfunctions, impairment in locomotor behavior, and neurodegeneration [34–37].

Finally, genetic screens have identified mutations in genes that regulate lifespan in *Drosophila*, although they are not distinct components of characterized signaling pathways. A P-element mutant line for the G-protein coupled receptor methuselah was isolated and shown to confer an approximately 35% increase in average lifespan and they exhibited increased ability to resist various stresses [38]. Also, the *Drosophila* gene *stunted*, which is the ligand for *methuselah* and forms the epsilon-subunit of mitochondrial ATP synthase, was found to extend lifespan and confers maternal effects [39]. In particular, *Drosophila* embryos lacking maternal *stunted* show defects in actin furrow formation, spindle orientation, nuclear divisions, and centrosome positioning in the cortical divisions [40]. Reduced activity of the gene *I’m not dead yet* (*Indy*), which encodes a transporter of Krebs cycle intermediates, was originally shown to double the average lifespan of flies without a loss of fertility or physical activity. It was postulated that Indy extends lifespan by triggering changes in metabolism [41]. However, the results for *Indy* were later considered controversial [42].

Silent information regulator 2 (Sir2 or Sirtuin) proteins are a class of evolutionary conserved proteins that share highly conserved enzymatic core domains [43]. They have been established as crucial players in mediating multiple physiological processes in humans and model organisms, including aging and longevity [44]. In *Drosophila*, dSir2 acts as a NAD-dependent deacetylase and catalyzes the deacetylation of lysine residues in core histone tails [45]. Previous work has shown that dSir2 regulates lifespan in flies [46]. In particular, ubiquitous overexpression of dSir2 using the UAS-Gal4 system increased lifespan by 18%–57%, while neuronal overexpression extends lifespan by 20%–52% [47]. It was further suggested that dSir2 and dietary restriction are controlled via a common pathway to modulate lifespan in flies. This conclusion was based on results showing that dietary restriction up-regulated *dSir2* transcription but it did not prolong lifespan in flies overexpressing dSir2, and lifespan extension by dietary restriction was inhibited in dSir2 deficient flies [47,48]. However, it was recently reported that standardization of genetic background and the use of appropriate controls abolished the increased longevity effect of dSir2 in *Drosophila*, and that dietary restriction increased fly lifespan independently of dSir2 [49]. In addition, lifespan extension by dietary restriction leads to increase in expression of dSir2 and a simultaneous decrease in expression of the histone deacetylase Rpd3 [48].

*Drosophila* Sir2 and Rpd3 are members of a more complex pathway that also includes the tumor suppressor protein p53 [46]. This protein responds to a variety of stresses that affect cell homeostasis [50,51]. Previous research has demonstrated a close association between p53 and the Igf-TOR signal transduction pathways in mammals [52]. Recent evidence from studies in *Drosophila* and other model organisms has further suggested that p53 may alter aging and longevity under certain conditions [53]. In particular, *p53* null *Drosophila* mutants or flies expressing the dominant-negative version of *p53* in muscle or fat body tissues demonstrated a shorter lifespan compared to wild-type controls, while transgenic flies overexpressing the dominant-negative *p53* in neurons displayed a longer lifespan. Interestingly, when the latter flies were dietary restricted, they showed a similar lifespan compared to dietary restricted wild-type control flies [54]. These results supported that p53 and dietary restriction are parts of a common pathway that regulates longevity in *Drosophila*. Furthermore, it was shown that expression of dominant-negative *p53* in the brain of the adult fly is sufficient to inhibit insulin signaling and extends lifespan [55].

The c-Jun *N*-terminal kinase (JNK) pathway is a pleiotropic intracellular signaling cascade that is involved in critical biological outcomes such as proliferation, differentiation, morphogenesis, apoptosis, and immunity [56]. Previous results also indicate a regulatory crosstalk between the JNK and NF-κB pathways in *Drosophila* [57]. JNK transduces signals from cell-surface receptors to the nucleus in response to various stresses, via the sequential phosphorylation of a series of kinases [58]. JNK signaling has recently been implicated in lifespan regulation in *Drosophila* [59]. In particular, JNK has been shown to extend lifespan by controlling repair processes and provide protection against various damages or stresses. For instance, mutant flies with enhanced JNK signaling accumulate less oxidative damage and live longer than wild-type control flies [60]. In addition, JNK affects lifespan by interacting with the IIS complex. Previous studies have demonstrated that JNK prevents IIS activity, and JNK overexpression increases *Drosophila* lifespan in a dFOXO-dependent manner [61,62].

The expression of certain heat-shock proteins (Hsps) is controlled by the JNK signaling pathway and the transcription factor *FOXO*. Activation of JNK extends lifespan in a FOXO-dependent manner and reduces IIS [61]. Hsps are molecular chaperones, a specialized set of proteins that are synthesized in response to thermal stress. They bind to unfolded proteins and promote protein folding and degradation [63]. Previous research has implicated Hsps in the regulation of lifespan in *Drosophila*; however, the evidence for the role of Hsps in longevity is currently incomplete [64]. Induced overexpression of various Hsps has been found to increase fly lifespan. For example, transgenic flies overexpressing *Hsp70* at low levels were shown to be resistant to heat and displayed prolonged lifespan at normal temperatures [65], although a different study failed to verify this observation [66]. Overexpression of small *Hsp26* and *Hsp27* in tissue-general pattern confers higher resistance to stress and increased lifespan to flies [67]. However, overexpression of the small mitochondrial *Hsp22* specifically in motor neurons also rendered flies resistant to stresses and extended lifespan, whereas decreased expression of this Hsp had the opposite effects [68]. Such results in *Drosophila*, as well as in other invertebrate models, emphasize that genetic perturbation of Hsp expression reveals the connection between proteostasis and age-related diseases and the aging process.

Aging cells gradually accumulate malfunctioning or damaged cytosolic components, resulting in cellular function decline that ultimately leads to cell death and disease. Autophagy is a highly conserved pathway that maintains cell homeostasis and an efficient stress response by degrading intracellular deleterious materials. Autophagy deficiency during aging is considered the main cause for the detrimental effects imposed on cells due to reduced protection against damage [69]. Recent work in invertebrate models suggests that loss-of-function in essential autophagy genes accelerates aging and lifespan shortening in *Drosophila* [70]. Flies with defective autophagy-related genes *Atg7* and *Atg8a* have decreased lifespan and earlier symptoms of cellular aging [71,72]. Also, mutations in certain autophagic genes identified from a genetic screen result in reduced fly lifespan and changes in ubiquitin signaling pathways [73]. Remarkably, the polyamine spermidine has been recently reported to act as an inducer of autophagic activity that promotes longevity in flies [74]. In terms of tissue-specific effects of autophagy, reduced expression of several autophagy genes in *Drosophila* neurons has been observed with age, and elevating expression of *Atg8* in the fly brain rescues aged cells from accumulating dysfunctional mitochondria and extends longevity by at least 50% [72].

## 3. Immune Signaling Pathways in *Drosophila*

*Drosophila* has emerged as a paradigm for investigating innate immunity and also as a model for studying the mode of infection of human pathogens [75,76]. Previous and recent studies in the fruit fly have led to the identification of receptors for pathogen recognition, the molecular signaling pathways that regulate the downstream responses against the pathogens, and the activation of effector mechanisms employed to eliminate the infection [77]. The *Drosophila* immune system consists of two kinds of responses. First, the systemic immune response, which involves the production and secretion of antimicrobial peptides (AMP) and other immune factors by the fat body (equivalent to the mammalian liver) into the hemolymph (equivalent to mammalian blood) that fills the open circulatory system; and second, the acute-phase immune response that is activated by inflammatory cytokines and is responsible for the removal of foreign microbes by phagocytes or the complement defense mechanism [78].

The hallmark of the *Drosophila* host defense is the definition of two regulatory signaling pathways, Toll and Immune deficiency (Imd), which lead to the activation of distinct members of the Nuclear Factor kappa B (NF-κB) family of transcription factors, and result in the expression of hundreds of target genes, including those encoding AMP [79]. The Toll signaling pathway is activated mainly by fungi and Gram-positive bacteria and in some cases by Gram-negative bacteria, whereas the Imd pathway is mostly activated by Gram-negative bacteria. The Toll- and Imd-mediated systemic immune response plays a crucial role for resistance to infections that can occur upon host septic injury [80].

The Toll pathway requires the tight regulation of an extracellular cascade involving proteases that activate PRR and an intracellular cascade involving NF-κB elements that induce the transcription of target genes. In particular, the transmembrane receptor Toll is activated upon binding a cleaved form of the extracellular cytokine Spaetzle that is processed by proteolytic cascades. These are initiated by upstream secreted recognition molecules, such as peptidoglycan-recognition proteins (PGRP) and Gram-negative-bacteria-binding proteins (GNBP) or by direct cleavage of serine proteases by fungal virulence factors. Toll activation (dimerization) results in the recruitment of the sorting adaptor MyD88 [81], and the signaling adaptors Tube and Pelle (intracellular death domain-containing proteins) that cause the phosphorylation and proteasomal degradation of the *Drosophila* inhibitory κB (IκB) homolog, Cactus. This leads to the release and translocation of the Rel transcription factors *Dorsal* and *Dorsal-related Immune Factor* (*Dif*) into the nucleus where they bind NF-κB response elements and activate the transcription of AMP related genes, like *Drosomycin* [82]. The finding that the Toll pathway is implicated in the systemic immune response of *Drosophila* paved the way for the discovery of Toll-like Receptors (TLR) as PRR in mammals [83]. The importance of this discovery was recognized in the 2011 Nobel Prize in Physiology or Medicine [84].

The *Drosophila* Imd signaling pathway comprises several components that have counterparts functioning in the Tumor Necrosis Factor (TNF) pathway in mammals [85]. This pathway is activated upon direct binding of PGRP-LC to diaminopimelic acid-containing peptidoglycan (DAP-PGN) of Gram-negative bacteria. PGRP-LC recruits the scaffold protein Imd that in turn brings together the adaptor protein dFADD and the caspase Dredd. This pathway also involves activation of the IκB kinase complex (Ird5 and Kenny) by TAK1 that depends on its adaptor protein TAB2, as well as on Imd and possibly dFADD and DIAP2. This cascade of molecular events leads to phosphorylation and cleavage of the ANKyrin repeats of Relish that results in the translocation of the Rel domain to the nucleus where it binds NF-κB response elements and activates the transcription of AMP related genes, like *Diptericin* [86,87].

Although the Toll and Imd pathways can be induced separately, they can also function together. Depending on the type of microbial challenge, both immune cascades can be induced at different levels in response to septic injury in *Drosophila* adult flies. Co-regulation between the Toll and Imd pathways often occurs at the transcriptional level or at the gene promoter level through the involvement of different κB-responsive elements from each immune pathway. Cross-talk between Toll and Imd pathways results in the production of several peptides and proteins upon direct injection of microbes into the hemocoel (insect body cavity), which leads to amplification of the systemic immune response of the host against the pathogen [88]. Various such molecules have recently been identified, but their specific contribution to the *Drosophila* immune response still remains unclear. In addition, two other pathways, it has also been suggested that Janus Kinase/Signal Transducer Activator of Transcription (JAK/STAT) and JNK take part in the systemic immune response by acting in a competing or cooperative mode [89]. Finally, it has been previously shown that the Imd components TAB2/TAK1 also trigger activation of JNK signaling, whereas negative feedback can occur between the Imd-Relish and Imd-JNK branches of the *Drosophila* immune system [90].

## 4. Relationship between Aging and Immunity in *Drosophila*

Changes in the transcription of genes encoding antibacterial peptides and proteins have been reported in aged *Drosophila*. A previous study found lower levels of *Diptericin* transcripts in aged flies upon inoculation with heat-killed bacteria compared to young flies [91]. However, higher transcription levels of *Diptericin* were found in aged flies than in young flies after challenge with live bacteria. The authors proposed that aged flies have impaired immune responses, such as phagocytosis and melanization, and are therefore less able to clear bacterial infections. As a result, prolonged persistence of bacteria in aged flies leads to extended induction of pathways leading to antimicrobial peptide production, such as the Imd pathway, and increased generation of *Diptericin* transcripts. Moreover, expression of AMP-GFP transgenic constructs was partially predictive of the remaining lifespan in young flies, and pharmacological inhibition of NF-κB prolongs lifespan of *Drosophila* [92,93]. Transcriptomic analysis of aging flies kept under full-nutrient conditions and low-calorie conditions has shown a significant increase in transcription of five *PGRP* genes (*PGRP-LA*, *PGRP-LB*, *PGRP-LC*, *PGRP-SA* and *PGRP-SC1b*) in aging flies in both treatments [94]. In addition, increased transcription levels of several antimicrobial effector genes such as *Cecropins*, *Attacins* and *Defensin*, as well as *Relish*, which is a key factor in the induction of the *Drosophila* humoral response, were present in aging flies [87]. Another independent transcriptomic study that looked at spatio-temporal regulation of gene expression in tissues of adult flies also found that *PGRP-LC* expression increases considerably at the middle of the adult stage and that *Diptericin* expression increases in flies of advanced age [95]. In particular *PGRP-LC* expression in young flies was shown to be limited to a small number of cells in sensory organs of the head and thorax, and in aged flies higher expression was observed in the oenocytes. These results further emphasize the increased activity of immune related genes during aging in *Drosophila*. Interestingly, it has been shown that *Methuselah* transcription is reduced in *Dorsal* mutant flies, which implies that the Toll pathway transcriptional factor *Dorsal* regulates expression of *Methuselah* [96], and therefore it may act in two ways in adult *Drosophila* by affecting aging and controlling the expression of antimicrobial peptides.

Estimating the ability of *Drosophila* to survive pathogenic infection over time and pathogen load in infected flies are important aspects to determine resistance/tolerance phenotypes and elucidate the contribution of signaling pathways to the immune response [97]. It has previously been shown that older flies are less able to suppress the growth of *Escherichia coli* following infection [98]. A study that aimed to correlate immune activation, pathogen resistance, and aging reported that acute overexpression of *PGRP-LE* in the fat body of *Drosophila* adult flies is sufficient to induce strong up-regulation of antimicrobial peptides and enhanced resistance to infection by the gram-negative bacterium *Pseudomonas aeruginosa* and the gram-positive *Enterococcus faecalis* and *Staphylococcus aureus* without causing any significant effects in various fitness traits [99]. However, *PGRP-LE* overexpression for the duration of the adult stage significantly reduces lifespan that is due to continuous activation of *Relish*. These results suggest that reduced lifespan is not the outcome of immune function deterioration in these flies, but the result of chronic inflammation. The effect of bacterial species occurring in fly cultures has formerly been studied. Presence of *Wolbachia* in fly stocks influences longevity but not the immune response [100–102]. Other bacteria found in *Drosophila* laboratory cultures also enhance longevity when they are present during the first week of adult life, but their impact on the immune response of the fly appears to be complex [103]. A more recent work investigated the effect of bacterial burden on *Drosophila* lifespan and found that aerobic and anaerobic bacterial burden inside the body and on the surface of adult flies increases strongly with age, but lifespan is not affected by bacterial burden [104]. The authors concluded that flies carrying a significant number of microbes are still able to trigger a robust immune response without a cost to lifespan. Simultaneous examination of the ability of *Drosophila* male and female adult flies to survive infection with different numbers of bacteria as well as clear the infection across age indicated that survival of infected flies decreases during aging but bacterial elimination remains unaffected through age [105]. More recently, flies pre-subjected to a cold treatment and fed on a diet lacking live yeast were tested for their ability to resist infection by the entomopathogenic fungus *Beauveria bassiana*. The results revealed that flies kept in the absence of yeast have reduced resistance to fungal infection, and this adverse effect on resistance can be compensated by a cold pretreatment of the flies [106]. The mechanism that leads to the cold induced tolerance to fungal infection is currently unknown. It is speculated that this effect could be due to increased production of antimicrobial peptides or up-regulation of other immune responses in cold-pretreated flies; however, these hypotheses need to be proven experimentally [107].

The *Drosophila* cellular immune response is less well characterized than the humoral response. Cellular immunity in flies involves the action of circulating hemocytes (equivalent to mammalian white blood cells) that participate in several immune functions such as cell spreading, cell aggregation, formation of nodules, phagocytosis and encapsulation of foreign invaders [108]. The effectiveness of the cellular immune response in old flies was recently investigated by examining the numbers and activity of hemocytes in male and female wild-type adult flies of different ages [109]. The data showed a substantial decrease in hemocyte numbers in older female, but not male, flies compared to younger individuals. Also, phagocytosis (microbial engulfment) of fluorescent *E. coli* bacterial cells or *B. bassiana* fungal spores by hemocytes declined with the age of the flies. These results denote that the cellular arm of the *Drosophila* immune function deteriorates significantly in aged flies.

The IIS pathway has been found to modulate lifespan, stress and pathogen resistance in *Drosophila*. Flies with mutations conferring increased lifespan (*puckered* or *puc* in the JNK signaling pathway and *Chico* in insulin signaling pathway) as well as dietary restricted flies were tested for their ability to survive infection with different pathogens and activate the transcription of antimicrobial peptide genes in response to pathogenic challenge [110]. It was shown that *puc* heterozygous and *Chico* homozygous and heterozygous mutant flies have improved survival after infection with gram-positive or gram-negative bacteria, but there is no effect of diet restriction on pathogen resistance. Increased survival of *puc* and *Chico* mutants is not due to up-regulation of AMP, since no significant differences in mRNA levels of *Diptericin*, *Drosomycin* and *Attacin* are found between mutant and wild-type control flies after infection with *P. aeruginosa* or *E. faecalis* (Figure 1). More surprisingly, stronger expression of antimicrobial peptides in dietary restricted flies is not accompanied with improved resistance to the bacteria. Finally, the authors failed to confirm the hypothesis that *dFOXO* activation plays a role in the protection to the pathogens. However, a recent study reports that *dFOXO* transcription factor regulates antimicrobial gene expression (Figure 1) [111]. In particular, it is shown that *dFOXO* is able to bind to the AMP gene regulatory region to activate AMP transcription in uninfected flies as well as in infected flies with defective Toll and Imd pathways. In terms of tissue-specificity, stimulation of the Toll pathway during infection prevents the action of IIS on subcellular localization of *dFOXO* in the fat body of the fly; it initiates the accumulation of *dFOXO* in the nucleus and therefore its activation [112]. These studies point out a connection between innate immunity and key regulators of the IIS pathway in *Drosophila*.

Genetic studies to identify changes in the function of the innate immune system of young and old fly lines has found significant variation in the immune response, as measured for clearance of *E. coli* infection, between lines at each age group. However, there was no genetic correlation of immune function across ages, which indicates that different genetic loci are responsible for the variation in immune response at each age [113]. Examination of allelic variation at *S6 kinase* (*S6k*), which is part of the TOR pathway, has identified *S6k* alleles that confer pleiotropic effects on various phenotypic features related to metabolism and fitness, including lifespan and immune function [114]. More recently, microarray analysis to determine the transcriptional response of young and aged inbred wild-type *Drosophila* lines to infection and wounding has revealed a strong correlation between variation in gene transcription with clearance of *E. coli* bacteria in aged flies compared to young flies. Bioinformatic analysis has shown that several of the identified genes are involved in clearance of *E. coli* bacteria in aged flies, and they are associated with energy metabolism [115]. In particular, *fatty acid synthase*, *gonadotropin-releasing hormone receptor* (an adipokinetic hormone-binding gene), and *RAC serine/threonine-protein kinase* (interacts with other proteins to regulate the insulin signaling and TOR pathways, inhibits the activation of FOXO, and is involved in modulating the expression of antimicrobial peptides) showed a positive correlation (higher expression level was associated with higher infection level—weak bacterial clearance). In contrast, *mos* (a kinase that promotes positive regulation of TOR signaling) showed a negative correlation (higher expression level was associated with lower infection level—strong bacterial clearance) [115].

To investigate the role of autophagy in lifespan extension and immune response in *Drosophila*, conditional RNA interference has previously been used to silence the autophagy genes *Atg5*, *Atg7* and *Atg12* in adult flies [116]. Inactivation of these *Atg* genes results in reduced survival of flies to *E. coli* infection and increased bacterial load, but has no effect on lifespan of uninfected flies. The outcomes of these experiments illustrate that proper function of *Atg* genes is an important factor for efficient immune response in *Drosophila* adults and that dysfunctional autophagy does not affect fly lifespan. Finally, knockdown of the expression of the gene *dSUR*, which encodes the regulatory subunit of K_ATP_ potassium channels, increases the lethality of *Drosophila* after infection with the cardiotropic Flock House virus [117], and more recent genetics and pharmacological studies have revealed that K_ATP_ channels are able to control the viral load in the fly heart during an infection and therefore they constitute an important antiviral resistance mechanism in *Drosophila* [118]. Previous work has shown that *dSUR* expression decreases with aging, and that this decrease in *dSUR* expression is associated with increased pacing-induced heart failure [119].

## 5. Major Challenges and Future Perspectives

Promising research directions in the field of aging and immunity in *Drosophila* are currently focusing on the identification and analysis of evolutionary conserved pathways that affect both lifespan and immune function. For instance, it is currently unknown whether Hsps or related proteins act as modulators of both aging processes and immune resistance pathways to infection in flies. Future studies using tissue-specific and developmental stage-specific changes in Hsps will be particularly useful for characterizing potential immune mechanisms for their effects on specific times during the adult stage and tissues for their action. Since the effect of Hsps on the lifespan of female flies has not been examined so far, and because sex-specific effects have been reported to affect lifespan and immunity in *Drosophila* [120,121], it would be interesting to test whether results for the Hsps on male flies are in concert with those obtained for female individuals. In addition, transcription factor *dFOXO* has been shown to control the expression of the Hsp *l(2)efl* (“essential for life”), the overexpression of which increases fly lifespan and since JNK pathway activation increases lifespan in a dFOXO-dependent manner [61], further work is needed to identify the involvement of *l(2)efl* in lifespan extension by JNK, its potential role in the immune response, and the molecular mechanisms through which these processes are controlled in *Drosophila*.

Interestingly, most studies investigating the interplay between aging and immune mechanisms in *Drosophila* as well as in other model organisms have conducted experiments using various pathogenic or non-pathogenic bacterial species. It will be equally important to comprehensively assess the effects of *Drosophila* immune responses to viral and fungal pathogens as well as to parasitic organisms and whether/how these reactions associate with lifespan extension mechanisms. These studies should not be limited to analyzing changes in the expression of target genes in immune signaling pathways but they should further examine whether expression of immune recognition molecules is altered with aging and how this affects the overall ability of the host to mount an immune response and resist infection.

Recent efforts in *Drosophila* immunology research have expanded to the diagnosis of “cause of death” of infected flies as well as the identification and characterization of pathological effects imposed by various pathogens on the host [122]. Given that studies aimed at eliciting the relationship between immunity and aging in *Drosophila* need to consider lifespan measurement with tissue-specific effects detrimental to the fly, this area of research would benefit from studies on mutant strains with maximized longevity to characterize the molecular events underlying the physiological factors that cause organ failure under infectious conditions, thereby leading to reduced lifespan.

Another interesting aspect of future research will be to compare the efficiency of immune responses between laboratory wild-type *Drosophila* strains and flies collected from the field. It is expected that insects in their natural environments, in contrast to those kept in nearly sterile laboratory environments, will have their immune systems constitutively activated due to chronic exposure to a range of pathogenic organisms and interactions with their intra- and extra-cellular microbial symbionts. Changes in immune function in natural *Drosophila* populations could likely be linked to aging mechanisms that would affect fly longevity. Regarding gut microflora in flies, it will be of particular interest to correlate the presence or absence of certain symbiotic microbes in the gut with lifespan extension ability and resistance to artificially delivered pathogenic microorganisms. Similarly, identification of the mechanisms by which *Wolbachia* endosymbionts influence aging and are protected from immune system provocation are exciting areas of future investigation.

Finally, previous studies in model organisms have shown that most long-lived mutants are resistant to one or several stresses; however, certain mutant combinations are able to separate aging mechanisms and defenses to various stresses, including infection by pathogenic microbes [123]. Therefore, it will be crucial that future research will explore the impact of immune signaling pathways on processes that affect lifespan in *Drosophila* through positive or negative interaction with intracellular and extracellular pathogens. Alternatively, future studies will focus on the role of other mechanisms, like autophagy, necrosis and apoptosis, which are potentially involved in co-regulating these important biological functions. Such work will lay the foundation for the identification of additional molecular/genetic pathways that may play key roles not only in *Drosophila* but also in vertebrate organisms, perhaps even in humans.

## 6. Conclusions

The availability of powerful genetics and genomics tools in *Drosophila* has significantly contributed toward the identification and characterization of genes which are controlled by universal signaling pathways. These pathways are required for the activation of the immune response against pathogenic infections and the regulation of aging mechanisms that determine lifespan. A high priority for this exciting field of research will be to determine whether changes in immune capacity of *Drosophila* aging mutants are associated with functional deterioration of particular organs or tissues. Research in model systems, such as *Drosophila*, will continue to collect evidence for the interaction between immunity and aging mechanisms, and whether altering one process affects the other. Furthermore, studies using long-lived mutants of model organisms in immunity research will help to identify key players involved in the regulation of the immune response in vertebrate animals. In terms of human health, understanding the relationship between aging and immune system function is of critical importance, particularly as the average human lifespan lengthens, increasing the impact of age-related diseases.
